# Ethidium benzoate methanol monosolvate

**DOI:** 10.1107/S241431462400302X

**Published:** 2024-04-18

**Authors:** Runa Shimazaki, Masaaki Sadakiyo

**Affiliations:** a Department of Applied Chemistry, Faculty of Science Division I, Tokyo University of Science, 1-3 Kagurazaka, Shinjuku-ku, Tokyo, 162-8601, Japan; Sunway University, Malaysia

**Keywords:** crystal structure, ethidium salt, benzoate, hydrogen bonding

## Abstract

In the title salt solvate (systematic name: 8-amino-5-ethyl-6-phenyl­phenanthridin-5-ium benzoate methanol monosolvate), C_21_H_20_N_3_^+^·C_6_H_5_CO_2_^−^·CH_3_OH, two ethidium cations, C_21_H_20_N_3_^+^, dimerize about a twofold axis through π–π inter­actions [inter-centroid separation = 3.6137 (4) Å]. The benzoate anions are connected through hydrogen bonding with the –NH_2_ groups of the ethidium cations and the –OH group of the MeOH mol­ecule. The MeOH mol­ecule also accepts a hydrogen bond from the –NH_2_ group of the ethidium cation. The result is a one-dimensional hydrogen-bonded chain along the *b-*axis direction.

## Abstract 2

The crystal structure of a new ethidium salt has been determined. Two ethidium cations self-associate to form a dimer through π–π inter­actions.

## Graphical abstract

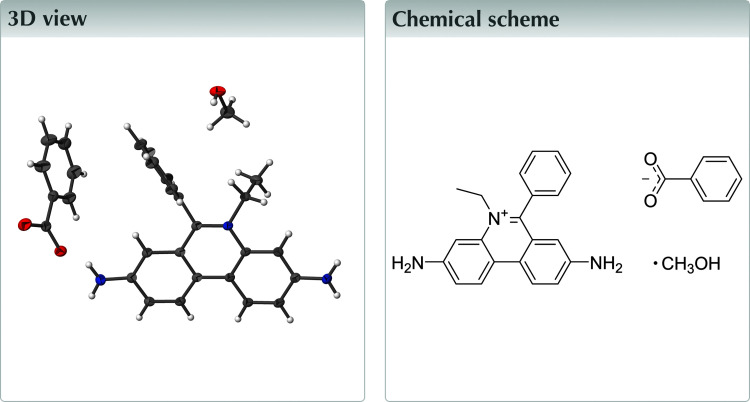

## Structure description

**Figure 1:**
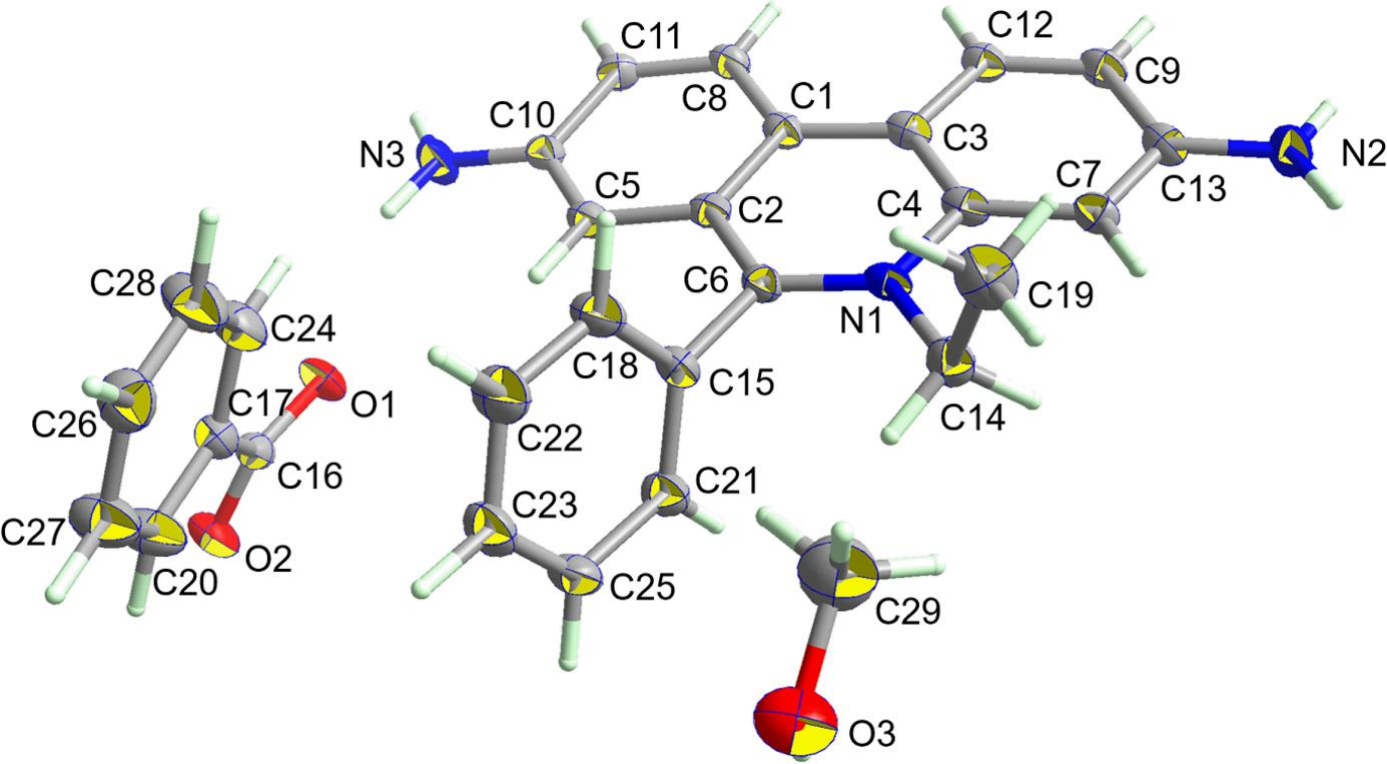
The structure of the crystallographically independent mol­ecules in C_21_H_20_N_3_^+^·C_6_H_5_CO_2_^−^·CH_3_OH with displacement ellipsoids drawn at the 50% probability level.

**Figure 2:**
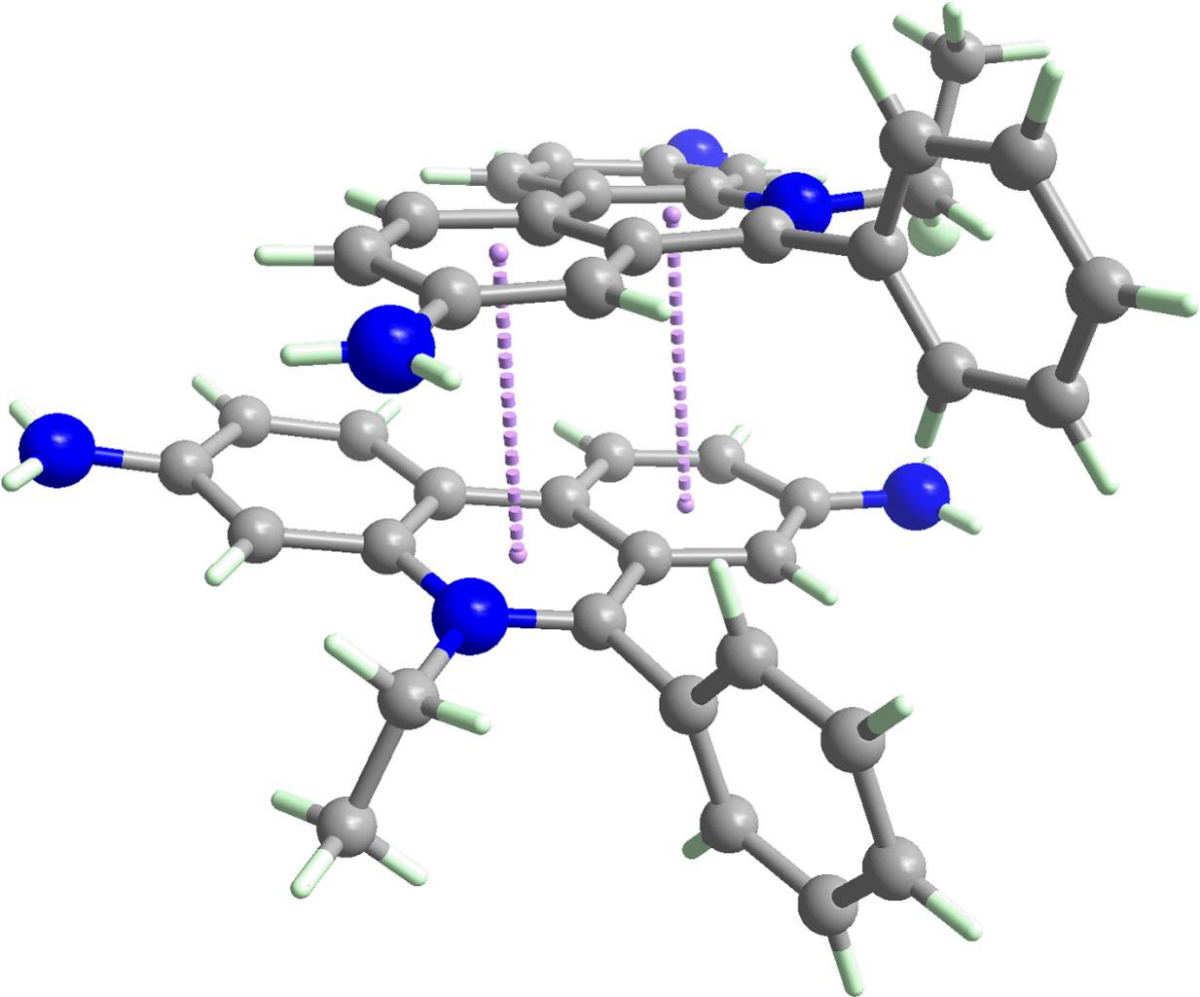
The dimer formed by two ethidium cations. The *Cg*⋯*Cg* separations [inter-centroid separation: 3.6137 (4) Å] are indicated as purple dotted lines.

**Figure 3:**
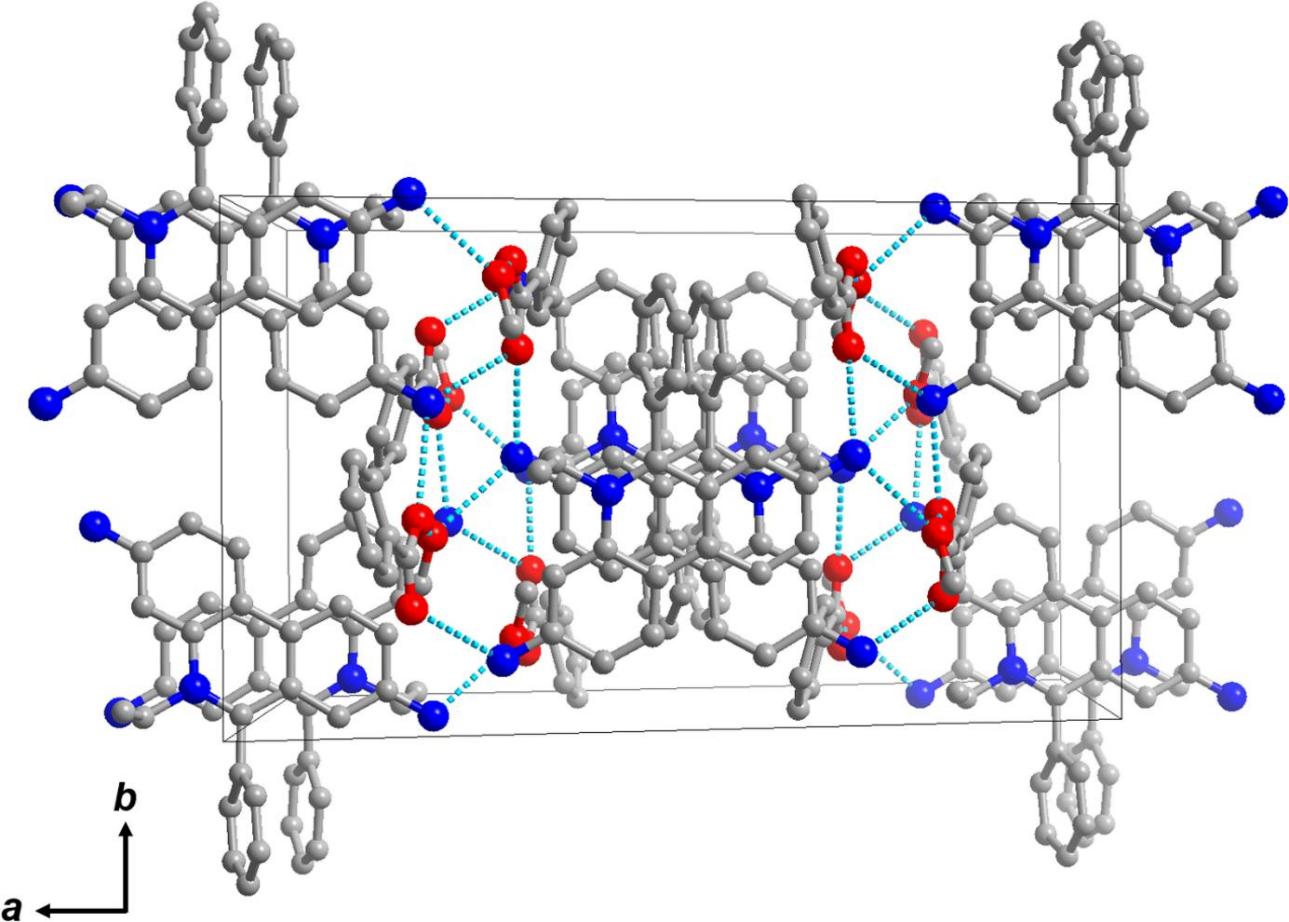
An illustration of the hydrogen-bonding network. Hydrogen bonds are shown as dotted lines.

Ethidium salts have various applications such as an inter­calator for DNA (Chen *et al.*, 2000 ▸) and as a building block for covalent organic frameworks (Ma *et al.*, 2016 ▸). In this study, the structure of a new ethidium salt solvate, C_21_H_20_N_3_^+^·C_6_H_5_CO_2_^−^·MeOH, is reported (Fig. 1 ▸). The dihedral angle between the pendant ring and the fused ring system is 77.01 (6)°. Two ethidium cations associate about a twofold axis *via* π–π stacking (Fig. 2 ▸). The closest separation between the mol­ecular planes is approximately 3.4 Å [the *Cg*⋯*Cg* separation is 3.6137 (4) Å], indicating the presence of π–π inter­actions. The three components of C_21_H_20_N_3_^+^·C_6_H_5_CO_2_^−^·CH_3_OH are connected by hydrogen bonds (Table 1 ▸) formed along the *b-*axis direction, resulting in the formation of a one-dimensional hydrogen-bonded chain (Fig. 3 ▸).

## Synthesis and crystallization

An aqueous solution (120 ml) of silver(I) benzoate (45.8 mg 0.20 mmol) was mixed with an aqueous solution (200 ml) of ethidium bromide (78.9 mg, 0.20 mmol) and then the mixture was stirred overnight at room temperature. After that, the precipitate was removed by centrifugation. The remaining solution was evaporated to obtain a crude powder. The powder was dissolved in methanol (4 ml) and the remaining precipitate was again removed by centrifugation. By evaporation of the remaining solution, a red powder was obtained. Red single crystals of [C_21_H_20_N_3_][C_6_H_5_CO_2_]·MeOH were obtained by slow evaporation (for 11 days) of a solution of the powder (7 mg) dissolved in 1 ml of a mixed solvent system, H_2_O/MeOH (1:1).

## Refinement

Details of crystal data, data collection and structure refinement are given in Table 2 ▸.

## Supplementary Material

Crystal structure: contains datablock(s) I. DOI: 10.1107/S241431462400302X/tk4103sup1.cif

Structure factors: contains datablock(s) I. DOI: 10.1107/S241431462400302X/tk4103Isup2.hkl

Supporting information file. DOI: 10.1107/S241431462400302X/tk4103Isup3.cdx

Supporting information file. DOI: 10.1107/S241431462400302X/tk4103sup4.txt

Supporting information file. DOI: 10.1107/S241431462400302X/tk4103Isup5.cml

CCDC reference: 2347501

Additional supporting information:  crystallographic information; 3D view; checkCIF report

## Figures and Tables

**Table 1:** Hydrogen-bond geometry (Å, °) Hydrogen-bond geometry (Å, °).

*D*—H⋯*A*	*D*—H	H⋯*A*	*D*⋯*A*	*D*—H⋯*A*
O3—H3⋯O2^i^	0.84	1.85	2.6636 (17)	163
N2—H2⋯O1^ii^	0.88	2.09	2.8741 (18)	148
N2—H2*A*⋯O2^iii^	0.88	2.06	2.9271 (17)	169
N3—H3*A*⋯O1	0.88	2.18	2.9264 (18)	142
N3—H3*B*⋯O3^iv^	0.88	2.09	2.939 (2)	162

Symmetry codes: (i) 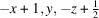 ; (ii) 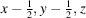 ; (iii) 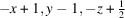 ; (iv) 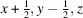 .

**Table 2:** Experimental details

Crystal data	
Chemical formula	C_21_H_20_N_3_^+^·C_7_H_5_O_2_^−^·CH_4_O
*M*_r_	467.55
Crystal system, space group	Monoclinic, *C*2/*c*
Temperature (K)	90
*a*, *b*, *c* (Å)	22.0407 (6), 12.4642 (3), 18.0706 (5)
β (°)	107.4952 (10)
*V* (Å^3^)	4734.7 (2)
*Z*	8
Radiation type	Mo *K*α
μ (mm^−1^)	0.09
Crystal size (mm)	0.30 × 0.15 × 0.10

Data collection	
Diffractometer	Bruker PHOTON II CPAD
Absorption correction	Multi-scan (*SADABS*; Krause *et al.*, 2015 ▸)
*T*_min_, *T*_max_	0.691, 0.746
No. of measured, independent and observed [*I* > 2σ(*I*)] reflections	29963, 6087, 4736
*R*_int_	0.064
(sin θ/λ)_max_ (Å^−1^)	0.686

Refinement	
*R*[*F* ^2^ > 2σ(*F* ^2^)], *wR*(*F* ^2^), *S*	0.053, 0.150, 1.05
No. of reflections	6087
No. of parameters	319
H-atom treatment	H-atom parameters constrained
Δρ_max_, Δρ_min_ (e Å^−3^)	0.51, −0.27

Computer programs: *APEX4* and *SAINT* (Bruker, 2021 ▸), *SIR2019* (Burla *et al.*, 2015 ▸), *SHELXL2018/3* (Sheldrick, 2015 ▸), *DIAMOND* (Brandenburg, 2014 ▸) and *Yadokari-XG* (Kabuto *et al.*, 2009 ▸).

## full crystallographic data

### Crystal data

**Table:**

C_21_H_20_N_3_^+^·C_7_H_5_O_2_^−^·CH_4_O	*F*(000) = 1984
*M**_r_* = 467.55	*D*_x_ = 1.312 Mg m^−^^3^
Monoclinic, *C*2/*c*	Mo *K*α radiation, λ = 0.71069 Å
*a* = 22.0407 (6) Å	Cell parameters from 6092 reflections
*b* = 12.4642 (3) Å	θ = 2.8–28.6°
*c* = 18.0706 (5) Å	µ = 0.09 mm^−^^1^
β = 107.4952 (10)°	*T* = 90 K
*V* = 4734.7 (2) Å^3^	Block, red
*Z* = 8	0.30 × 0.15 × 0.10 mm

### Data collection

**Table:**

Bruker PHOTON II CPAD diffractometer	4736 reflections with *I* > 2σ(*I*)
Radiation source: fine-focus sealed tube	*R*_int_ = 0.064
φ and ω scans	θ_max_ = 29.2°, θ_min_ = 1.9°
Absorption correction: multi-scan (SADABS; Krause *et al.*, 2015)	*h* = −29→27
*T*_min_ = 0.691, *T*_max_ = 0.746	*k* = −16→15
29963 measured reflections	*l* = −24→24
6087 independent reflections	

### Refinement

**Table:**

Refinement on *F*^2^	0 restraints
Least-squares matrix: full	Hydrogen site location: inferred from neighbouring sites
*R*[*F*^2^ > 2σ(*F*^2^)] = 0.053	H-atom parameters constrained
*wR*(*F*^2^) = 0.150	*w* = 1/[σ^2^(*F*_o_^2^) + (0.0683*P*)^2^ + 4.3384*P*] where *P* = (*F*_o_^2^ + 2*F*_c_^2^)/3
*S* = 1.05	(Δ/σ)_max_ < 0.001
6087 reflections	Δρ_max_ = 0.51 e Å^−^^3^
319 parameters	Δρ_min_ = −0.27 e Å^−^^3^

### Special details

**Table:**

Geometry. All esds (except the esd in the dihedral angle between two l.s. planes) are estimated using the full covariance matrix. The cell esds are taken into account individually in the estimation of esds in distances, angles and torsion angles; correlations between esds in cell parameters are only used when they are defined by crystal symmetry. An approximate (isotropic) treatment of cell esds is used for estimating esds involving l.s. planes.
Refinement. Refinement of F^2^ against ALL reflections. The weighted R-factor wR and goodness of fit S are based on F^2^, conventional R-factors R are based on F, with F set to zero for negative F^2^. The threshold expression of F^2^ > 2sigma(F^2^) is used only for calculating R-factors(gt) etc. and is not relevant to the choice of reflections for refinement. R-factors based on F^2^ are statistically about twice as large as those based on F, and R- factors based on ALL data will be even larger. All hydrogen atoms are geometrically fixed using a riding-model approximation with C—H = 0.95 (for phenyl), 0.98 (for methyl), 0.99 (for methylene), O—H = 0.84 and N—H = 0.88 Å.

### Fractional atomic coordinates and isotropic or equivalent isotropic displacement parameters (Å^2^)

**Table:**

	*x*	*y*	*z*	*U*_iso_*/*U*_eq_	
N1	0.41329 (6)	0.45032 (9)	0.33925 (7)	0.0185 (3)	
O1	0.69273 (5)	0.72829 (8)	0.31166 (7)	0.0271 (3)	
C1	0.52679 (7)	0.33972 (11)	0.35270 (8)	0.0188 (3)	
C2	0.52645 (7)	0.45352 (11)	0.35874 (8)	0.0187 (3)	
C3	0.46802 (7)	0.28272 (11)	0.33491 (8)	0.0189 (3)	
O2	0.69837 (6)	0.89712 (9)	0.27351 (7)	0.0308 (3)	
C4	0.41035 (7)	0.33857 (11)	0.32620 (8)	0.0190 (3)	
C5	0.58308 (7)	0.51335 (11)	0.36914 (8)	0.0203 (3)	
H5	0.581926	0.589338	0.372557	0.024*	
C6	0.46801 (7)	0.50549 (11)	0.35457 (8)	0.0184 (3)	
O3	0.29255 (6)	0.86288 (10)	0.36827 (7)	0.0333 (3)	
H3	0.297556	0.860786	0.324012	0.050*	
N2	0.29253 (6)	0.12236 (10)	0.26776 (8)	0.0271 (3)	
H2	0.256774	0.157719	0.261451	0.033*	
H2A	0.291688	0.052819	0.259098	0.033*	
C7	0.35174 (7)	0.28527 (12)	0.30498 (9)	0.0209 (3)	
H7	0.313740	0.324333	0.300220	0.025*	
C8	0.58634 (7)	0.28949 (12)	0.36280 (8)	0.0209 (3)	
H8	0.588537	0.213396	0.361985	0.025*	
C9	0.40670 (7)	0.11801 (12)	0.30101 (9)	0.0230 (3)	
H9	0.405605	0.042653	0.292810	0.028*	
C10	0.64007 (7)	0.46248 (12)	0.37438 (8)	0.0206 (3)	
N3	0.69510 (7)	0.51611 (11)	0.38180 (9)	0.0291 (3)	
H3A	0.695708	0.586676	0.383493	0.035*	
H3B	0.730214	0.480361	0.384930	0.035*	
C11	0.64045 (7)	0.34775 (12)	0.37364 (8)	0.0217 (3)	
H11	0.679646	0.311216	0.380930	0.026*	
C12	0.46366 (7)	0.17005 (12)	0.32241 (9)	0.0213 (3)	
H12	0.501570	0.129871	0.329174	0.026*	
C13	0.34882 (7)	0.17493 (12)	0.29078 (8)	0.0213 (3)	
C14	0.35344 (7)	0.50599 (12)	0.33950 (9)	0.0218 (3)	
H14	0.317546	0.478366	0.296421	0.026*	
H14A	0.357563	0.583836	0.331271	0.026*	
C15	0.46851 (7)	0.62318 (11)	0.36976 (8)	0.0195 (3)	
C16	0.68832 (7)	0.82758 (12)	0.31927 (9)	0.0223 (3)	
C17	0.67009 (7)	0.86787 (12)	0.38850 (9)	0.0236 (3)	
C18	0.49149 (8)	0.66099 (13)	0.44535 (9)	0.0255 (3)	
H18	0.503879	0.611805	0.487379	0.031*	
C19	0.33973 (8)	0.48795 (14)	0.41613 (10)	0.0298 (4)	
H19	0.340212	0.410852	0.426940	0.045*	
H19A	0.297811	0.517464	0.412963	0.045*	
H19B	0.372324	0.523897	0.457891	0.045*	
C20	0.65307 (9)	0.97436 (14)	0.39351 (10)	0.0342 (4)	
H20	0.652014	1.022597	0.352441	0.041*	
C21	0.45042 (8)	0.69580 (12)	0.30839 (9)	0.0240 (3)	
H21	0.434456	0.670472	0.256566	0.029*	
C22	0.49639 (9)	0.77075 (13)	0.45959 (10)	0.0300 (4)	
H22	0.511866	0.796341	0.511400	0.036*	
C23	0.47885 (8)	0.84285 (13)	0.39869 (10)	0.0289 (4)	
H23	0.482578	0.917811	0.408518	0.035*	
C24	0.67216 (9)	0.79923 (14)	0.44987 (10)	0.0324 (4)	
H24	0.683412	0.726078	0.447166	0.039*	
C25	0.45578 (8)	0.80517 (13)	0.32318 (10)	0.0278 (3)	
H25	0.443564	0.854601	0.281290	0.033*	
C26	0.64006 (9)	0.94195 (15)	0.51899 (10)	0.0340 (4)	
H26	0.629493	0.967049	0.563207	0.041*	
C27	0.63763 (10)	1.01061 (15)	0.45818 (11)	0.0374 (4)	
H27	0.625279	1.083222	0.460647	0.045*	
C28	0.65795 (10)	0.83670 (15)	0.51494 (11)	0.0391 (4)	
H28	0.660563	0.789493	0.557056	0.047*	
C29	0.30916 (9)	0.76352 (14)	0.40501 (11)	0.0351 (4)	
H29	0.354249	0.749124	0.412005	0.053*	
H29A	0.283218	0.706941	0.372969	0.053*	
H29B	0.301619	0.764985	0.455754	0.053*	

### Atomic displacement parameters (Å^2^)

**Table:**

	*U*^11^	*U*^22^	*U*^33^	*U*^12^	*U*^13^	*U*^23^
N1	0.0208 (6)	0.0152 (6)	0.0197 (6)	0.0019 (4)	0.0063 (5)	0.0003 (4)
O1	0.0308 (6)	0.0187 (5)	0.0341 (6)	0.0025 (4)	0.0133 (5)	0.0023 (4)
C1	0.0236 (7)	0.0166 (7)	0.0165 (6)	0.0012 (5)	0.0064 (5)	0.0007 (5)
C2	0.0223 (7)	0.0171 (7)	0.0160 (6)	0.0022 (5)	0.0048 (5)	0.0008 (5)
C3	0.0224 (7)	0.0166 (7)	0.0188 (7)	0.0010 (5)	0.0077 (6)	0.0001 (5)
O2	0.0429 (7)	0.0208 (6)	0.0331 (6)	−0.0014 (5)	0.0181 (5)	0.0030 (4)
C4	0.0244 (7)	0.0146 (7)	0.0179 (6)	0.0012 (5)	0.0064 (5)	−0.0007 (5)
C5	0.0243 (7)	0.0152 (7)	0.0212 (7)	0.0007 (5)	0.0065 (6)	0.0011 (5)
C6	0.0234 (7)	0.0155 (7)	0.0162 (6)	0.0011 (5)	0.0056 (5)	0.0010 (5)
O3	0.0448 (7)	0.0262 (6)	0.0324 (6)	0.0095 (5)	0.0169 (6)	0.0010 (5)
N2	0.0233 (7)	0.0180 (6)	0.0405 (8)	−0.0020 (5)	0.0102 (6)	−0.0053 (5)
C7	0.0209 (7)	0.0184 (7)	0.0247 (7)	0.0018 (5)	0.0085 (6)	0.0003 (5)
C8	0.0241 (7)	0.0159 (7)	0.0220 (7)	0.0031 (5)	0.0057 (6)	−0.0001 (5)
C9	0.0289 (8)	0.0157 (7)	0.0254 (7)	0.0003 (6)	0.0099 (6)	−0.0015 (5)
C10	0.0222 (7)	0.0201 (7)	0.0194 (7)	0.0001 (5)	0.0061 (6)	0.0016 (5)
N3	0.0231 (7)	0.0198 (6)	0.0457 (9)	0.0012 (5)	0.0122 (6)	0.0026 (6)
C11	0.0218 (7)	0.0209 (7)	0.0225 (7)	0.0039 (5)	0.0066 (6)	0.0013 (5)
C12	0.0236 (7)	0.0174 (7)	0.0232 (7)	0.0023 (5)	0.0077 (6)	−0.0002 (5)
C13	0.0248 (7)	0.0192 (7)	0.0209 (7)	−0.0012 (5)	0.0085 (6)	−0.0014 (5)
C14	0.0209 (7)	0.0158 (7)	0.0294 (8)	0.0030 (5)	0.0086 (6)	−0.0010 (5)
C15	0.0195 (7)	0.0157 (7)	0.0237 (7)	0.0012 (5)	0.0069 (6)	−0.0008 (5)
C16	0.0204 (7)	0.0200 (7)	0.0260 (7)	0.0001 (5)	0.0062 (6)	0.0009 (6)
C17	0.0238 (7)	0.0222 (7)	0.0239 (7)	−0.0019 (6)	0.0059 (6)	0.0005 (6)
C18	0.0320 (8)	0.0208 (7)	0.0221 (7)	0.0018 (6)	0.0059 (6)	0.0005 (6)
C19	0.0331 (9)	0.0253 (8)	0.0361 (9)	0.0038 (6)	0.0178 (7)	−0.0020 (6)
C20	0.0497 (11)	0.0247 (8)	0.0302 (9)	0.0074 (7)	0.0153 (8)	0.0053 (7)
C21	0.0282 (8)	0.0194 (7)	0.0225 (7)	0.0026 (6)	0.0048 (6)	0.0007 (5)
C22	0.0408 (10)	0.0232 (8)	0.0240 (8)	−0.0001 (7)	0.0068 (7)	−0.0048 (6)
C23	0.0345 (9)	0.0161 (7)	0.0347 (9)	0.0008 (6)	0.0082 (7)	−0.0033 (6)
C24	0.0453 (10)	0.0225 (8)	0.0312 (9)	0.0001 (7)	0.0140 (8)	0.0025 (6)
C25	0.0335 (9)	0.0185 (7)	0.0296 (8)	0.0043 (6)	0.0070 (7)	0.0036 (6)
C26	0.0395 (10)	0.0354 (9)	0.0285 (8)	−0.0029 (7)	0.0125 (7)	−0.0045 (7)
C27	0.0518 (12)	0.0271 (9)	0.0335 (9)	0.0075 (8)	0.0133 (8)	−0.0013 (7)
C28	0.0572 (12)	0.0304 (9)	0.0322 (9)	−0.0039 (8)	0.0174 (9)	0.0040 (7)
C29	0.0366 (10)	0.0297 (9)	0.0391 (10)	0.0067 (7)	0.0117 (8)	0.0031 (7)

### Geometric parameters (Å, º)

**Table:**

N1—C6	1.3428 (19)	C12—H12	0.9500
N1—C4	1.4109 (18)	C14—C19	1.520 (2)
N1—C14	1.4917 (18)	C14—H14	0.9900
O1—C16	1.2523 (18)	C14—H14A	0.9900
C1—C8	1.416 (2)	C15—C18	1.389 (2)
C1—C2	1.423 (2)	C15—C21	1.394 (2)
C1—C3	1.427 (2)	C16—C17	1.510 (2)
C2—C5	1.417 (2)	C17—C20	1.390 (2)
C2—C6	1.423 (2)	C17—C24	1.390 (2)
C3—C4	1.416 (2)	C18—C22	1.390 (2)
C3—C12	1.421 (2)	C18—H18	0.9500
O2—C16	1.2626 (19)	C19—H19	0.9800
C4—C7	1.400 (2)	C19—H19A	0.9800
C5—C10	1.385 (2)	C19—H19B	0.9800
C5—H5	0.9500	C20—C27	1.387 (3)
C6—C15	1.4918 (19)	C20—H20	0.9500
O3—C29	1.401 (2)	C21—C25	1.387 (2)
O3—H3	0.8400	C21—H21	0.9500
N2—C13	1.353 (2)	C22—C23	1.383 (2)
N2—H2	0.8800	C22—H22	0.9500
N2—H2A	0.8800	C23—C25	1.387 (2)
C7—C13	1.397 (2)	C23—H23	0.9500
C7—H7	0.9500	C24—C28	1.386 (3)
C8—C11	1.359 (2)	C24—H24	0.9500
C8—H8	0.9500	C25—H25	0.9500
C9—C12	1.362 (2)	C26—C28	1.378 (3)
C9—C13	1.422 (2)	C26—C27	1.381 (3)
C9—H9	0.9500	C26—H26	0.9500
C10—N3	1.356 (2)	C27—H27	0.9500
C10—C11	1.430 (2)	C28—H28	0.9500
N3—H3A	0.8800	C29—H29	0.9800
N3—H3B	0.8800	C29—H29A	0.9800
C11—H11	0.9500	C29—H29B	0.9800
			
C6—N1—C4	122.23 (12)	C19—C14—H14A	109.5
C6—N1—C14	120.09 (12)	H14—C14—H14A	108.1
C4—N1—C14	117.61 (12)	C18—C15—C21	119.65 (14)
C8—C1—C2	117.20 (13)	C18—C15—C6	119.68 (13)
C8—C1—C3	123.55 (13)	C21—C15—C6	120.51 (13)
C2—C1—C3	119.23 (13)	O1—C16—O2	124.88 (15)
C5—C2—C1	120.66 (13)	O1—C16—C17	117.93 (13)
C5—C2—C6	120.96 (13)	O2—C16—C17	117.19 (13)
C1—C2—C6	118.38 (13)	C20—C17—C24	118.72 (15)
C4—C3—C12	116.91 (13)	C20—C17—C16	121.05 (14)
C4—C3—C1	120.27 (13)	C24—C17—C16	120.19 (14)
C12—C3—C1	122.78 (13)	C15—C18—C22	120.07 (14)
C7—C4—N1	120.44 (13)	C15—C18—H18	120.0
C7—C4—C3	121.39 (13)	C22—C18—H18	120.0
N1—C4—C3	118.17 (13)	C14—C19—H19	109.5
C10—C5—C2	120.85 (13)	C14—C19—H19A	109.5
C10—C5—H5	119.6	H19—C19—H19A	109.5
C2—C5—H5	119.6	C14—C19—H19B	109.5
N1—C6—C2	121.34 (13)	H19—C19—H19B	109.5
N1—C6—C15	119.87 (13)	H19A—C19—H19B	109.5
C2—C6—C15	118.77 (13)	C27—C20—C17	120.36 (16)
C29—O3—H3	109.5	C27—C20—H20	119.8
C13—N2—H2	120.0	C17—C20—H20	119.8
C13—N2—H2A	120.0	C25—C21—C15	119.84 (14)
H2—N2—H2A	120.0	C25—C21—H21	120.1
C13—C7—C4	120.35 (13)	C15—C21—H21	120.1
C13—C7—H7	119.8	C23—C22—C18	120.30 (15)
C4—C7—H7	119.8	C23—C22—H22	119.8
C11—C8—C1	121.44 (14)	C18—C22—H22	119.8
C11—C8—H8	119.3	C22—C23—C25	119.67 (15)
C1—C8—H8	119.3	C22—C23—H23	120.2
C12—C9—C13	120.90 (14)	C25—C23—H23	120.2
C12—C9—H9	119.5	C28—C24—C17	120.51 (16)
C13—C9—H9	119.5	C28—C24—H24	119.7
N3—C10—C5	123.17 (14)	C17—C24—H24	119.7
N3—C10—C11	119.10 (14)	C23—C25—C21	120.46 (15)
C5—C10—C11	117.71 (13)	C23—C25—H25	119.8
C10—N3—H3A	120.0	C21—C25—H25	119.8
C10—N3—H3B	120.0	C28—C26—C27	119.40 (17)
H3A—N3—H3B	120.0	C28—C26—H26	120.3
C8—C11—C10	121.88 (14)	C27—C26—H26	120.3
C8—C11—H11	119.1	C26—C27—C20	120.51 (17)
C10—C11—H11	119.1	C26—C27—H27	119.7
C9—C12—C3	121.90 (14)	C20—C27—H27	119.7
C9—C12—H12	119.1	C26—C28—C24	120.48 (17)
C3—C12—H12	119.1	C26—C28—H28	119.8
N2—C13—C7	121.36 (14)	C24—C28—H28	119.8
N2—C13—C9	120.13 (13)	O3—C29—H29	109.5
C7—C13—C9	118.51 (13)	O3—C29—H29A	109.5
N1—C14—C19	110.62 (12)	H29—C29—H29A	109.5
N1—C14—H14	109.5	O3—C29—H29B	109.5
C19—C14—H14	109.5	H29—C29—H29B	109.5
N1—C14—H14A	109.5	H29A—C29—H29B	109.5
			
C8—C1—C2—C5	4.2 (2)	C5—C10—C11—C8	4.5 (2)
C3—C1—C2—C5	−174.09 (13)	C13—C9—C12—C3	−0.4 (2)
C8—C1—C2—C6	−175.42 (12)	C4—C3—C12—C9	1.7 (2)
C3—C1—C2—C6	6.3 (2)	C1—C3—C12—C9	−176.06 (14)
C8—C1—C3—C4	179.54 (13)	C4—C7—C13—N2	−177.83 (14)
C2—C1—C3—C4	−2.3 (2)	C4—C7—C13—C9	2.2 (2)
C8—C1—C3—C12	−2.8 (2)	C12—C9—C13—N2	178.47 (14)
C2—C1—C3—C12	175.40 (13)	C12—C9—C13—C7	−1.5 (2)
C6—N1—C4—C7	−175.44 (13)	C6—N1—C14—C19	−98.78 (15)
C14—N1—C4—C7	7.59 (19)	C4—N1—C14—C19	78.27 (16)
C6—N1—C4—C3	4.3 (2)	N1—C6—C15—C18	103.00 (17)
C14—N1—C4—C3	−172.63 (12)	C2—C6—C15—C18	−75.35 (19)
C12—C3—C4—C7	−1.0 (2)	N1—C6—C15—C21	−81.64 (18)
C1—C3—C4—C7	176.79 (13)	C2—C6—C15—C21	100.00 (17)
C12—C3—C4—N1	179.18 (12)	O1—C16—C17—C20	−168.22 (16)
C1—C3—C4—N1	−3.0 (2)	O2—C16—C17—C20	12.2 (2)
C1—C2—C5—C10	−0.6 (2)	O1—C16—C17—C24	14.0 (2)
C6—C2—C5—C10	178.99 (13)	O2—C16—C17—C24	−165.61 (16)
C4—N1—C6—C2	−0.2 (2)	C21—C15—C18—C22	0.0 (2)
C14—N1—C6—C2	176.68 (12)	C6—C15—C18—C22	175.40 (15)
C4—N1—C6—C15	−178.53 (12)	C24—C17—C20—C27	−0.9 (3)
C14—N1—C6—C15	−1.6 (2)	C16—C17—C20—C27	−178.76 (17)
C5—C2—C6—N1	175.22 (13)	C18—C15—C21—C25	0.3 (2)
C1—C2—C6—N1	−5.2 (2)	C6—C15—C21—C25	−175.05 (14)
C5—C2—C6—C15	−6.5 (2)	C15—C18—C22—C23	−0.4 (3)
C1—C2—C6—C15	173.15 (12)	C18—C22—C23—C25	0.5 (3)
N1—C4—C7—C13	178.89 (13)	C20—C17—C24—C28	−0.5 (3)
C3—C4—C7—C13	−0.9 (2)	C16—C17—C24—C28	177.42 (16)
C2—C1—C8—C11	−3.5 (2)	C22—C23—C25—C21	−0.2 (3)
C3—C1—C8—C11	174.73 (14)	C15—C21—C25—C23	−0.2 (3)
C2—C5—C10—N3	177.79 (14)	C28—C26—C27—C20	−0.1 (3)
C2—C5—C10—C11	−3.7 (2)	C17—C20—C27—C26	1.2 (3)
C1—C8—C11—C10	−0.9 (2)	C27—C26—C28—C24	−1.3 (3)
N3—C10—C11—C8	−176.92 (14)	C17—C24—C28—C26	1.6 (3)

### Hydrogen-bond geometry (Å, º)

Hydrogen-bond geometry (Å, °).

**Table:**

*D*—H···*A*	*D*—H	H···*A*	*D*···*A*	*D*—H···*A*
O3—H3···O2^i^	0.84	1.85	2.6636 (17)	163
N2—H2···O1^ii^	0.88	2.09	2.8741 (18)	148
N2—H2*A*···O2^iii^	0.88	2.06	2.9271 (17)	169
N3—H3*A*···O1	0.88	2.18	2.9264 (18)	142
N3—H3*B*···O3^iv^	0.88	2.09	2.939 (2)	162

Symmetry codes: (i) −*x*+1, *y*, −*z*+1/2; (ii) *x*−1/2, *y*−1/2, *z*; (iii) −*x*+1, *y*−1, −*z*+1/2; (iv) *x*+1/2, *y*−1/2, *z*.

## References

[bb1] Brandenburg, K. (2014). *DIAMOND.* Crystal Impact GbR, Bonn, Germany.

[bb2] Bruker (2021). *APEX4* and *SAINT*. Bruker AXS Inc., Madison, Wisconsin, USA.

[bb3] Burla, M. C., Caliandro, R., Carrozzini, B., Cascarano, G. L., Cuocci, C., Giacovazzo, C., Mallamo, M., Mazzone, A. & Polidori, G. (2015). *J. Appl. Cryst.* **48**, 306–309.

[bb4] Chen, W., Turro, N. J. & Tomalia, D. A. (2000). *Langmuir*, **16**, 15–19.

[bb5] Kabuto, C., Akine, S., Nemoto, T. & Kwon, E. (2009). *J. Crystallogr. Soc. Japan*, **51**, 218–224.

[bb6] Krause, L., Herbst-Irmer, R., Sheldrick, G. M. & Stalke, D. (2015). *J. Appl. Cryst.* **48**, 3–10. 10.1107/S1600576714022985 PMC4453166 26089746

[bb7] Ma, H., Liu, B., Li, B., Zhang, L., Li, Y.-G., Tan, H.-Q., Zang, H.-Y. & Zhu, G. (2016). *J. Am. Chem. Soc.* **138**, 5897–5903. 10.1021/jacs.5b13490 27094048

[bb8] Sheldrick, G. M. (2015). *Acta Cryst.* C**71**, 3–8.

